# Bloodless bilateral lung transplant in a patient, a Jehovah's Witness, with pulmonary fibrosis and secondary pulmonary hypertension

**DOI:** 10.1016/j.xjtc.2026.102447

**Published:** 2026-06-03

**Authors:** Scott T. Vasher, Anila Khan, Kevin Tsui, Lisa Potter, Flor Cerda, Cynthia Serdiuk, Rozmeen Shivji, Lauren Doak, Rade Tomic, Pablo G. Sanchez

**Affiliations:** a Biological Sciences Division, University of Chicago, Chicago, Ill; b Pulmonary Division, Northwestern University, Chicago, Ill; c Department of Pharmacy, University of Chicago, Chicago, Ill; d Transplant Surgery, University of Chicago Medical Center, Chicago, Ill

**Figure:**
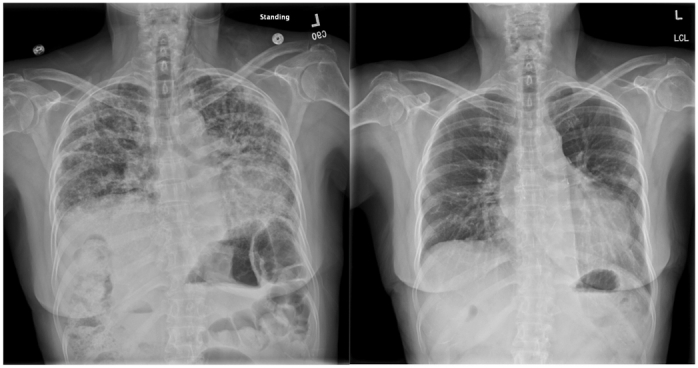
Chest radiographs from before and 9 months after lung transplant.

**Central Message**

Comprehensive perioperative optimization and a precise intraoperative low-dose heparin ECMO protocol enabled a bloodless bilateral lung transplant in a high-risk patient who was a Jehovah's Witness.

A 67-year-old woman with idiopathic pulmonary fibrosis and secondary pulmonary hypertension who was a Jehovah's Witness was referred to our center after being declined at several other institutions because of advanced age, frailty, and declining blood products. On presentation, she required 6 L of supplemental oxygen per minute and had a GAP (ie, Gender, Age, and Physiology) index of 7, corresponding to a 62% probability of death at 2 years.^1^ Her condition deteriorated, necessitating admission for heated high-flow nasal cannula support to provide 50% fraction of inspired oxygen at 50 L/min, nutritional optimization, and intensive preoperative rehabilitation as part of a structured bloodless transplantation protocol. Written informed consent for publication to report this case was obtained from the patient in this study. This report did not require institutional review board approval.

Preoperatively, our multidisciplinary team used a risk factor identification and optimization strategy, detailed in Tables 1 and 2, based on established bloodless heart and lung transplant protocols.^2^, ^3^, ^4^, ^5^ Although this patient did not accept whole blood products, she did accept blood fractions such as prothrombin complex concentrate and albumin as well as recirculation of her own blood products with a cell-salvage device. Our protocol respects the need to avoid traditional blood transfusions but does require patients to accept blood fractions to proceed with transplant. Her preoperative treatment included intravenous erythropoietin, iron sucrose, vitamin B12, and folate, with dosages tailored to the initial hemoglobin level. The results of her baseline iron studies revealed ferritin 341 ng/mL, iron 121 μg/dL, total iron binding capacity 272 μg/dL, and hemoglobin 12.6 g/dL with blood group O positive. On hospital day 21, with hemoglobin at 10.7 g/dL, a course of erythropoietin and iron sucrose was commenced. She received iron sucrose 200 mg intravenous every other day for 5 doses and epoetin alfa 15,000 units intravenous daily for 13 days, with a goal of achieving hemoglobin 12 to 14 g/dL. Erythropoietin therapy was stopped once the hemoglobin increased above 13 g/dL.

**Table 1:** Bloodless lung transplant patient risk factor identification and laboratory testing performed pretransplant to optimize risk during evaluation and waitlisting

Risk factors identification
Severe right ventricular dysfunction
Expectation for difficult explant
Long-term systemic anticoagulation
Baseline uncorrectable anemia or thrombocytopenia
Pretransplant testing
Iron studies
Vitamin B12
Folic acid

**Table 2:** Bloodless lung transplant waitlist risk reduction, optimization, and postoperative strategies to maintain stable blood counts

Stop antiplatelet agents at least 5 d before waitlisting
Consider stopping antifibrotics and anemia-inducing medications
Use pediatric tubes for phlebotomy
Treat iron deficiency (ferritin <45 or % saturation <20)
Iron sucrose 200 mg intravenous every 48 h for 5 doses
Consider ferrous sulfate 325 mg by mouth daily after intravenous iron
Treat vitamin B12 deficiency
Cyanocobalamin 1000 mcg by mouth daily
Treat folate deficiency
Folic acid 1 mg by mouth daily
Treat thrombocytopenia
Eltrombopag 50 mg daily
Give epoetin alfa as needed to achieve hemoglobin 12-14 g/dL
Inpatient: 300 units/kg intravenous daily or 500 units/kg subcutaneously every 48 h
Admitted from home: 40,000 units intravenous once preoperatively

The patient completed her transplant evaluation and was listed for bilateral lung transplant on hospital day 39 with a composite allocation score of 34.5515-44.5515 and a hemoglobin of 14.8 g/dL on the day of listing. She was transplanted on hospital day 63, at which time her hemoglobin was 15.0 g/dL and her composite allocation score had increased to 43.6215-53.6215. Her donor was 150 cm tall (recipient: 152 cm), male, blood group O, with blunt head trauma as a cause of death.

During transplantation, central venoarterial extracorporeal membrane oxygenation (ECMO) was initiated, using an institutionally developed hybrid circuit capable of conversion to cardiopulmonary bypass, although this was not needed.^3^ Our intraoperative management strategy is shown in Table 3. Central aortic cannulation (18 Fr) and central right atrial venous cannulation (32 Fr angled) were used. Anticoagulation followed a single-shot low-dose heparin protocol: a bolus of 75 units/kg unfractionated heparin was administered to reach activated clotting time (ACT) ≥180 seconds; an additional 20 units/kg was given only if the target ACT was not achieved. No further ACT measurements or doses were required, and no protamine reversal was given postprocedure.^3^ Tranexamic acid was infused at 2 mg/kg/h after ECMO initiation and throughout the procedure for antifibrinolytic support. Blood conservation used cell salvage, with 220 mL of autologous blood reinfused postoperatively. Intraoperative crystalloid administration was restricted to 3.5 to 5.5 L, with albumin as the preferred volume expander to minimize dilutional anemia. Hemodynamic goals included mean arterial pressure >65 mm Hg, pulmonary artery pressures <35 mm Hg, and ECMO flow at 50% of calculated cardiac output, with a minimum perfusion rate of 2.0 L/min to maintain graft pulsatility.^3^ The total ischemia time was 308 minutes for the left lung and 395 minutes for the right lung.

**Table 3:** Bloodless lung transplant intraoperative management

Use cell-saving technique and devices for autotransfusion
Limit crystalloid to 3.5-5.5 L
Favor colloid over crystalloid for intraoperative volume expansion
Bolus 75 units/kg unfractionated heparin to achieve activated clotting time >180 s, rebolus 20 units/kg as needed
Tranexamic acid infusion at 2 mg/kg/h after ECMO initiated
Hemodynamic goals on ECMO
Mean arterial pressure >65 mm Hg
Pulmonary artery pressure <35 mm Hg
ECMO flow 50% of calculated cardiac output (minimum 2 L/min)

*ECMO*, Extracorporeal membrane oxygenation.

In our program, typical blood transfusion thresholds follow evidence-based standards and include marked hemodynamic compromise, uncontrollable bleeding, or hemoglobin <7 g/dL. This patient never met these criteria. If a patient required and was planned for a bloodless transplantation were to meet typical criteria for transfusion, their wishes not to receive blood would be respected. Instead, we would use volume expansion with albumin or hemostatic treatments if indicated such as prothrombin complex concentrate.

Postoperatively, blood draws were minimized and only performed as clinically indicated using pediatric tubes, further reducing iatrogenic anemia.^2^ Our postoperative management protocol matches our preoperative optimization strategy, shown in Table 2. This patient received erythropoietin 15,000 units intravenous daily for 3 days, iron sucrose 200 mg intravenous once, and ferrous sulfate 325 mg by mouth every 48 hours for a target hemoglobin ≥10 g/dL. Albumin remained the primary agent for volume resuscitation. Her nadir hemoglobin was 8.5 g/dL on postoperative day 2, and 9.0 g/dL on discharge (postoperative day 16). Her postoperative course was uncomplicated, with primary graft dysfunction grade 0 at 0, 24, 48, and 72 hours. She was extubated on postoperative day 2 and weaned to room air on postoperative day 7. She was discharged on postoperative day 15, and her total hospital length of stay was 78 days. Immunosuppression consisted of tacrolimus, mycophenolic acid, and prednisone. No further hematinic support was needed after discharge, and she rapidly regained baseline functional status. Four months posttransplant, she had returned to preoperative activity levels, and 8 months posttransplant her forced expiratory volume in 1 second had improved to 74% predicted from 24% pretransplant. She had no major complications in her posttransplant recovery.

This case demonstrates that using contemporary perioperative optimization, including anemia correction, low-dose heparin venoarterial ECMO support, cell salvage, and rigorous postoperative management, enabled true bloodless bilateral lung transplantation in a high-risk, frail patient who was a Jehovah's Witness.^2^^,^^3^^,^^5^ One limitation to building evidence in support of bloodless lung transplant is publication bias: unsuccessful or complicated cases of bloodless transplantation may be underreported. Future work in support of bloodless bilateral lung transplant could include case series of all cases within one or several institutions, including negative outcomes, to more fully characterize risk factors and outcomes. Multidisciplinary collaboration, patient consent for plasma fractions and supportive products, and strict adherence to blood conservation strategies were key to success. This experience highlights advances from bloodless cardiac transplant literature and institutional research that are applicable to complex cases in lung transplantation.^2^^,^^3^

## Conflict of Interest Statement

P.G.S. receives funding from the National Institutes of Health (R21-HL164436 and R01-HL174570). All other authors reported no conflicts of interest.

The *Journal* policy requires editors and reviewers to disclose conflicts of interest and to decline handling or reviewing manuscripts for which they may have a conflict of interest. The editors and reviewers of this article have no conflicts of interest.

## References

[1] Ley B., Ryerson C.J., Vittinghoff E. (2012). A multidimensional index and staging system for idiopathic pulmonary fibrosis. Ann Intern Med.

[2] Hozain A.E., Johnson B., Leiva O. (2026). Bloodless heart transplantation in patients who refuse transfusion: an 11-year case series. J Thorac Cardiovasc Surg.

[3] Chan E.G., Deitz R.L., Ryan J.P. (2025). Bloodless lung transplantation: comparison between 2 central venoarterial extracorporeal membrane oxygenation anticoagulation strategies and their impact on lung transplant outcomes. J Thorac Cardiovasc Surg.

[4] Tanaka A., Ota T., Uriel N. (2015). Cardiovascular surgery in Jehovah’s Witness patients: the role of preoperative optimization. J Thorac Cardiovasc Surg.

[5] Chan E.G., Morrell M.R., Chan P.G., Sanchez P.G. (2021). Bilateral sequential lung transplantation in Jehovah’s Witnesses. Perfusion.

